# Migrainous Infarction and Cortical Spreading Depression

**DOI:** 10.15190/d.2020.9

**Published:** 2020-08-12

**Authors:** Waleed Iftikhar, Fatima Fayyaz Cheema, Sneha Khanal, Qudsia Umaira Khan

**Affiliations:** ^1^CMH Lahore Medical College and Institute of Dentistry (NUMS), Lahore, Pakistan; ^2^Jahurul Islam Medical College and Hospital, Bajitpur, Kishoregonj, Bangladesh

**Keywords:** Migrainous infarction, cortical spreading depression, migraine, migraine with aura

## Abstract

Migraine is a very common disorder of the nervous system. It shares similar physiological processes with stroke. Migrainous infarction is a rare complication of migraine with aura. The neuro-logical symptoms of migraine aura correspond to the cortical spreading depression and this depression can lead to a migrainous infarction. It is pertinent to state that the investigation and detection of the cortical depression might have a great clinical significance. Blood vessels in the cranium play an important role in the pathophysiology of migraine.  In the case of injured states of brain, the cortical spreading depression causes extreme vasoconstriction rather than vasodilation. The endothelial damage caused by the cortical spreading depression can result in hypercoagulability, leading to an increased risk of stroke. There are many genetic disorders in which migraine and stroke are the major symptoms and an insight into these disorders can help us in the understanding of complex mechanisms of migrainous infarction. It is pertinent to state that some derangements in the vascular function accompany migraine which may also serve as targets for research and treatment. This article will describe the hemodynamic and genetic relationship between migraine induced stroke and how it relates to the cortical spreading depression.

## SUMMARY

*1. Introduction*

*2. Pathology and etiology of cortical spreading depression*

*3. Signs and symptoms of migrainous infarction*

*4. Diagnosis of migrainous infarction*

*5. The link between migrainous infarction and cortical spreading depression*

- *5.1. Hemodynamic implications*

- *5.2. Coagulation disorders*

- *5.3. Genetic implications*

*6. Treatment*

*7. Directions for future research*

*8. Conclusion*

## 1. Introduction

Migraine is a complex neurovascular disorder. It affects about one fifth of the population, among which 20-30% people experience sensory, motor and visual symptoms along with headache, a condition called as the migraine aura^1^. Migraine can progress through four stages, which are prodrome, aura, attack and post-drome, respectively. However, it is not necessary that everyone who has migraine goes through all these stages. Prodrome is the beginning of a migraine attack occurring few days or few hours before the migraine. The symptoms experienced during this phase include: mood changes, cravings for specific foods, fatigue, yawning, and sensitivity to light or sound. The prodromal phase can be followed by the aura phase in which the patient usually suffers from visual disturbances and numbness or tingling on one side of the body, symptoms that can last for 5-60 minutes. The third phase, the “attack”, also known as the “headache phase”, is characterized by stabbing pain either affecting one or both sides of the head along with sensitivity to light, nausea and anxiety and the symptoms lasting for 4-72 hours. The trigemino-vascular pathway is considered to be responsible for the debilitating headache observed in this phase. Post-drome, also called the “migraine hangover”, the last phase of migraine, is characterized by fatigue, lack of concentration, and neck stiffness, occurring in almost 80% of the patients and lasting for about 24-48 hours^2-4^.

Migraines are most probably due to brain ischemia, vascular pathologies or neurophysiological events e.g. cortical spreading depression. The same migraine-causing mechanisms may induce an infarction, potentially through a vasospasm. A migrainous infarction is a rare type of an ischemic stroke which occurs in correspondence with migraine aura symptoms. In migrainous strokes, the migraine must occur with aura. Migrainous strokes are rare and account for only 0.8 percent of all strokes. Typically, women under the age of 45 who experience migraine with aura are at the greatest risk for developing migrainous infarction, especially when combined with smoking and use of oral contraceptives. The symptoms of migraine aura can mimic the symptoms of a stroke, which is a leading cause of death across the world. According to the World Health Organization (WHO) about 15 million people have stroke worldwide each year. Evidences abound as to the relationship between migraine and vascular disorders, subclinical lesions of the brain and cortical depression. There are various mecha-nisms that may play a role in the link between cerebrovascular disorders and migraine^5-7^.

This review covers the pathology and etiology of cortical spreading depression, signs and symptoms and diagnosis of migrainous infarction. It highlights the link between migrainous infarction and cortical spreading depression through hemo-dynamic implications, coagulation disorders and genetic implications. It also briefly describes the treatment for migraine induced stroke and the directions for future research.

## 2. Pathology and etiology of cortical spreading depression

Studies show that the mechanisms causing the migraine can lead to an infarction. Cortical spreading depression is a neurophysiological event causing migraine which can also induce an infarction, potentially through a vasospasm.

In cortical spreading depression, there is the depression of spontaneous and evoked electro-encephalogram (EEG) activity. This depression spreads across the cortical surface at the rate of 2 mm to 5 mm. The marked depression of spontaneous EEG lasts for about 30 seconds to a minute, returning to the normal in 5 to 10 minutes. Conversely, the evoked synaptic activity takes at least 15 to 30 minutes to recover^8,9^. Propagating field oscillations precedes the neuronal and glial depolarization, which is spreading, covering up to 1 mm in distance^10^.A small state of hyper-excitability is indicated by these oscillations, which may relate to the observation of cortical spreading depression and seizures, in the same patients with acutely injured cerebral cortex^11^. A total loss of neuronal activity follows the oscillations, which may last for minutes, before complete recovery. At the same time, director current (DC) potential becomes negative with an amplitude within the range of 15 to 30 mV for 60 seconds^12^. Completely sustained depolarization may attempt to explain the negative shift in DC potential which is restricted to the unique cell domains, because at first, there is a rapid opening of conductance along a greater part of the pyramidal neuron accompanied by a centripetal closure in the direction of the apical dendrites^13^. There may also be some contribution from local increases in tissue resistivity^14^. Hence, it is possible that neurons may play a role in the current signals that initiate cortical spreading depolarization, and also for those that play a role in its propagation and determination^15^.

The depression of the activity seen with the EEG is caused by an acute failure of homeostasis of brain ions, and efflux of amino acids from the neurons, which is in some way similar to the ischemic penumbra^16,17^. During cortical spreading depression, there is an increase in the concentration of potassium ions in the extracellular fluid, up to 60 mmol/L from 30 mmol/L. Conversely, calcium ions concentration decreases from 0.1 mmol/L to 0.2 mmol/L, while chloride concentration decreases from 120 mmol/L to 70 mmol/L^18,19^. At the same time, there is a decline in pH from 7.3 to ~ 6.9, while there is also a 50% decrease in extracellular space dimensions to help regulate the movement of water into the neurons^20^.Movement of water into the dendrites results in reversible swelling of the neurons and alteration of dendritic spines while astrocyte volume remains constant^21-23^. This is in line with the involvement of neurons in cortical spreading depression. There is the spontaneous normalization of concentration of ions and extracellular space dimensions after 30 seconds to a minute, but the concentration of calcium ions and the extracellular pH (pH_e_) takes more time to recover.

A large concentration of amino acids, including aspartate and glutamate are released during the wave of depolarization^24,25^. Voltammetric recordings have indicated that the large release of transmitter accompanies the onset of depolarization^26^.This implies that the release of neurotransmitters may only play a secondary role in the spread of cortical spreading depression. Currently, we are yet to understand the mechanism by which cortical spreading depression is initiated. However, slight elevation in the level of potassium ions and transmitters are enough to start the spread of cortical spreading depression^27^.

**Figure 1:**
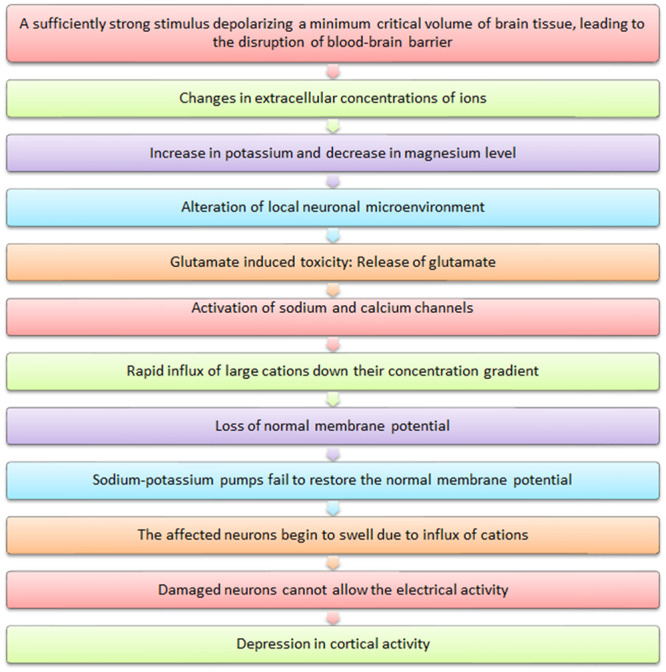
Mechanism of cortical spreading depression^28,29^

Cortical spreading depression is explained through the following flowchart (Figure 1)^28,29^.

## 3. Signs and symptoms of migrainous infarction

A person having a stroke or infarction during the course of migraine with aura is said to have migrainous infarction. The symptoms which are observed in patients of migraine with aura are most commonly visual symptoms such as flashes of light. However, sensory, language, and motor symptoms are also observed. These symptoms include dys-arthria (which may or may not be present), hyperactivity, hypoactivity, depression, cravings for specific food items, frequent yawning, fatigue, neck stiffness and pain, numbness or tingling on one side of the body or face, lack of concentration, sensitivity to light, sound, nausea and pallor. The symptoms of migraine with aura have been listed in the Table 1 below^30^.

**Table 1:** Symptoms of migraine with aura

Symptoms of migraine with aura
• Hyperactivity
• Hypoactivity
• Depression
• Cravings for specific food items
• Frequent yawning
• Fatigue
• Neck stiffness
• Neck pain
• Aphasia
• Dysarthria may or may not be present
• Visual disturbance
• Flashes of light
• Numbness or tingling on one side of face/body/tongue
• Difficulty in concentration
• Sensitivity to light
• Sensitivity to sound
• Nausea
• Pallor

Whenever blood supply to the brain is compromised, the brain cells and tissues start dying due to lack of supply of oxygen and nutrients. The symptoms of an ischemic stroke are dizziness, weakness or numbness especially on side of the body, severe headache, aphasia, visual disturbance, dysarthria, impaired consciousness, nystagmus and ataxia. The symptoms of an ischemic stroke have been listed in the Table 2 below^31,32^.

**Table 2:** Symptoms of an ischaemic stroke

Symptoms of an ischaemic stroke
• Dizziness
• Weakness, especially on one side of the body
• Numbness, especially on one side of the body
• Severe headache
• Visual disturbance
• Aphasia
• Dysarthria
• Impaired consciousness
• Nystagmus
• Ataxia

Most of the symptoms of migraine with aura and an ischemic stroke are overlapping. The major symptom of a migrainous infarction is visual aura (82.3%), closely accompanied by aphasia and sensory dysfunction. Symptoms that are also present include aphasia, mild hemiparesis, tetraparesis, and deficit in the visual field^31^.

## 4. Diagnosis of migrainous infarction

The International Classification of Headache Disorders (ICHD) is the basis for diagnosis^33^. Based on the ICHD criteria, at least 60-minute aura attack and premorbid migraine with aura diagnosis is necessary for the diagnosis of migrainous infarction.

An observation of an ischemic infarction using different neuroimaging techniques, such as Magnetic Resonance Imaging (MRI) and Computed Tomography Scan (CT Scan) in the migraine associated area adds confirmation to the diagnosis. Migrainous infarction occurs more frequently in the posterior circulation (70.6%-82.0%) as compared to the anterior circulation.

It is very difficult to differentiate between migraine with aura and a stroke, because symptoms of migraine with aura can mimic the symptoms of a stroke. However, one distinct feature that distinguishes the two is that the symptoms of migraine with aura develop gradually over a time of at least five minutes, while in the case of a stroke the symptoms are very sudden. A person with migraine with aura could have experienced the same symptoms multiple times. However, in the case of a stroke the symptoms are new for the person.

In many cases, the prognosis is usually favorable, exhibiting complete recovery or just a few residual symptoms, which may be very minor^34,35^.

## 5. The link between migrainous infarction and cortical spreading depression

Ischemic strokes occur when blood vessels to the brain are blocked, restricting blood flow. Cortical spreading depression, which is considered to be the underlying cause of migraine with aura, can lead to extreme vasoconstriction in case of injured states of brain. Thus, blood supply to the brain is compro-mised, leading to an infarction. Secondly, few studies suggest that cortical spreading depression damages the endothelium, which can result in hypercoagulability. This can also contribute to the chances of an infarction. There are many genetic disorders related to migraine and stroke. Better insights can help us in better understanding the migraine and its complex diversities.

### 5.1. Hemodynamic implications

It has been widely accepted that cortical spreading depression serves as a major pathogenic mechanism of migrainous infarction. The aura phase is the starting point of cerebral oligemia, it spreads interiorly in a gradual fashion, accompanied by hyperemia in an attack of migraine aura^36,37^. Patients without migraine aura also exhibit posterior hypoperfusion in an attack, as indicated by Positron Emission Tomography (PET) and MR perfusion studies^38,39^. When there is an inversion of the hemodynamic response to cortical spreading depression, mostly in pathological conditions, then the spreading depression may trigger extreme vasoconstriction rather than vasodilation^40^. However, the incidence of migrainous infarction is quite rare notwithstanding the high prevalence of the disorder, hence cerebral blood flow even at the oligemic phase of cortical spreading depression stands above the ischemic threshold. Alongside the oligemia itself, animal models of cortical spreading depression have shown ipsilateral release of matrix metalloprotease induced by depolarization, and subsequently, compromised integrity of the blood brain barrier^41^. There is also increased matrix metalloprotease in human migraineurs, especially during headache-free periods and in migraine attacks. When combined, cortical spreading depression, which is a special pathophysiologic mechanism of migrainous infarction, may trigger an ischemic stroke.

**Figure 2:**
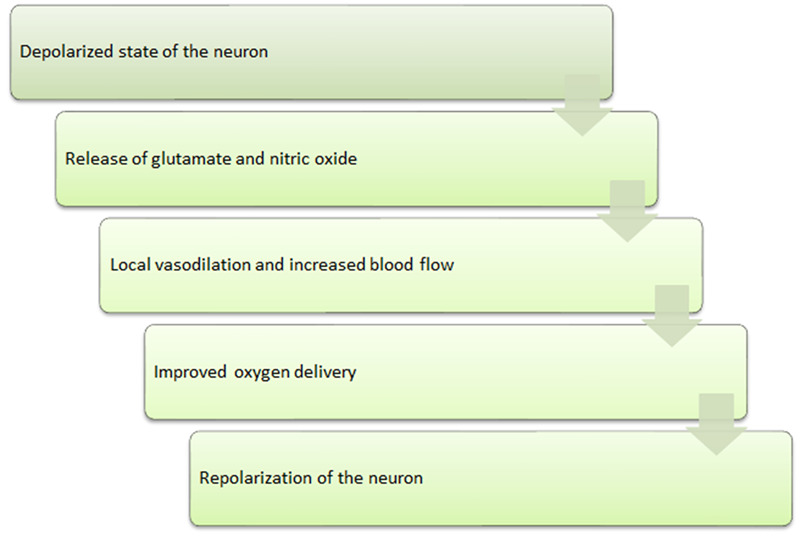
Vascular response to cortical spreading depression in the normal state of the brain^42^

**Figure 3:**
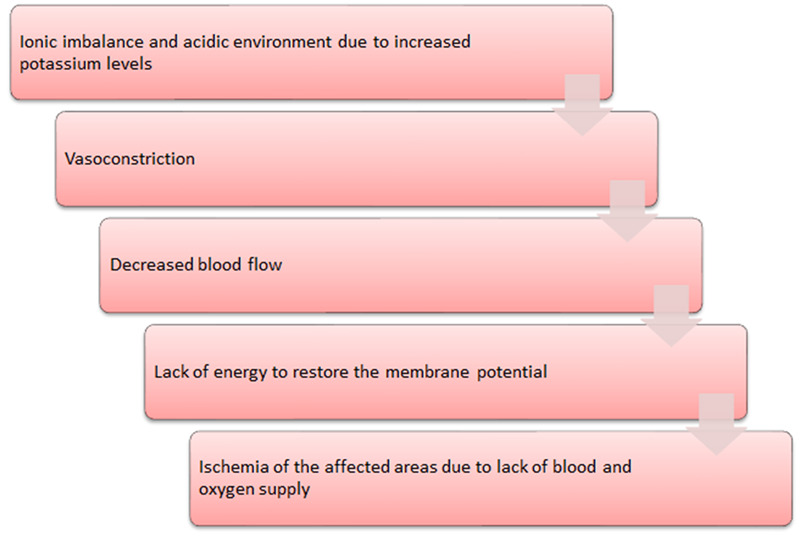
Vascular response to cortical spreading depression in the injured states of the brain^42^

Vascular response to cortical spreading depression in a healthy brain and in a damaged brain is described in the Figure 2 and Figure 3^42^.

### 5.2. Coagulation disorders

Studies have shown increased levels of Von Willebrand factor (VWF), platelet-activating factor and platelet aggregation during migraine episodes^43-45^. Platelet-activating factor is released from platelets, mast cells, and cerebral endothelial cells in response to calcitonin gene-related peptide and hypoxia. This in turn triggers the release of VWF. It is the VWF that activates the IIIa and IIb platelet receptors. Coagulation abnormalities include an increased level of prothrombin fragment 1.2, the deficiency of Protein S, and a reduced resistance to activated protein C^46,47^.

According to a study higher levels of endo-thelial micro particles (EMPs), platelets, red blood cells (RBCs), VWF antigen, fibrinogen, tissue plasminogen and activator antigen are reported in female patients with migraine with aura while monocytic and platelet micro particles are increased in all patients with migraine with aura. Stromal cell-derived factor-1 alpha, which is responsible for maintaining the integrity of the endothelium is deficient in women with migraine with aura. All these factors are responsible for causing hyper-coagulability, increasing the risk of stroke in patients with migraine with aura. However, we are yet to understand whether plasma hypercoagulability results from or is the cause of cortical spreading depression. One theory says that cortical spreading depression can cause endothelial damage. Thus, we can infer that hypercoagulability caused by this endothelial damage can lead to an infarction^48^.

### 5.3. Genetic implications

Cortical spreading depression presents the waves of depolarized glial cells and neurons propagating in a slow pattern accompanied by a sustained suppression of spontaneous activities in the neurons.

Imaging studies of patients suffering migraine have shown acute changes in brain activity and blood flow with features similar to those of cortical spreading depression, involved in visual aura migraine. Although the meninges are innervated by trigeminal nerves and plays a role in the genesis of migraine headaches, the mechanisms by which migraine is triggered remains controversial and not well understood. Studies on animal models have shown that the trigeminovascular afferents are activated by cortical spreading depression and evokes a series of events in the brainstem and cortical meninges that are consistent with the development of headaches^49^. Three familial hemiplegic migraines have been identified. These encode ion transporters, implying that alterations in neurotransmitter and ion balances in the brain play a role in this migraine type. Other monogenic syndromes, such as retinal vasculopathy with cerebral leukodystrophy (RVCL), cerebral autosomal dominant arteriopathy with subcortical infarcts and leukoencephalopathy (CADSIL) may give some molecular insight into the patho-physiology of migraine. There have been convincing replications of many genetic associations with potential migraine genes, such as methylene-tetrahydrofolate reductase (*MTHFR*), insulin receptor (*INSR*) and estrogen receptor 1 (*ESR1*). Different syndromes and diseases which have association with migraine, stroke and migraine-induced stroke are described in the Table 3 ^50-52^. There are several genes reported to be rarely mutated in case of the hemiplegic migraine (HM). These are *PRRT2, PNKD, SLC2A1, SLZ1A3*, and *SLC4A4*. These genes have been listed in the Table 4^51, 53^.

**Table 3:** Migraine and stroke related disorders and their associated genes and clinical presentations^50-52^

Disease	Gene Mutation	Clinical Presentation
Cerebral autosomal-dominant arteriopathy with subcortical infarcts and leukoencephalopathy (CADASIL)	Notch Receptor 3 (NOTCH3)	Migraine with aura; Encephalopathy; Seizures; Motor weakness.
Cerebral autosomal recessive arteriopathy with subcortical infarcts and leukoencephalopathy (CARASIL)	HtrA Serine Peptidase 1 (HTRA1)	Lacunar infarcts; Presence of non-neurological symptoms such as spondylosis and baldness.
Retinal vasculopathy with cerebral leukodystrophy (RVCL)	Three Prime Repair Exonuclease 1 (TREX1)	Migraine; Visual disturbance; Mini strokes; Cognitive impairment.
Hereditary infantile hemiparesis, retinal arteriolar tortuosity and leukoencephalopathy (HIHRATL)	Collagen Type IV Alpha 1 Chain (COL4A1)	Variable features, including both neurogical and systemic symptoms; Occurring in young children and adults.
Mitochondrial encephalomyopathy, lactic acidosis and stroke-like episodes (MELAS)	Mitochondrially encoded tRNA leucine 1 (MT-TL1)	Found in children; Migraine with abdominal disturbances; Seizures; Hearing loss.
Familial hemiplegic migraine 1 (FHM1)	Calcium Voltage-Gated Channel Subunit Alpha1 A (CACNA1A)	Migraine with aura; Hemiparesis; Seizures; Memory loss; Coma.
Familial hemiplegic migraine 2 (FHM2)	ATPase Na+/K+ Transporting Subunit Alpha 2 (ATP1A2)	Migraine with aura; Hemiparesis; Seizures; Memory loss; Coma.
Familial hemiplegic migraine 3 (FHM3)	Sodium Voltage-Gated Channel Alpha Subunit 1 (SCN1A)	Migraine with aura; Hemiparesis; Seizures; Memory loss; Coma.
Mendelian Migraine with Aura	Potassium channel subfamily K member 18 (KCNK18)	Typical migraine with aura.
Episodic Ataxia type 2 (EA2)	Calcium Voltage-Gated Channel Subunit Alpha1 A (CACNA1A)	Migraine; Nystagmus; Loss of balance and coordination; Vertigo; Muscle weakness.
Spinocerebellar Ataxia type 6 (SCA6)	Calcium Voltage-Gated Channel Subunit Alpha1 A (CACNA1A)	Migraine; Nystagmus; Cerebellar atrophy; Tingling and burning; Disarthria.
Familial Advanced Sleep-Phase Syndrome 2 (FASPS2)	Casein Kinase 1 Isoform Delta (CSNK1D)	Migraine with aura; Abnormal circadian rhythms.
Retinopathy, optic nerve edema, splenomegaly, anhidrosis, and migraine headache (ROSAH) Syndrome	Alpha-protein kinase 1 (ALPK1)	Migraine; Splenomegaly; Ocular dysfunction; Heatstroke; Inability to perspire.
Paroxysmal Dyskinesia Disorders	Proline-rich transmembrane protein 2 (PRRT2) Paroxysmal Nonkinesiogenic Dyskinesia (PNKD) Solute Carrier Family 1 Member 3 (SLC1A3)	Hemiplegic migraine; Attacks of involuntary movements.
Collagen Type IV Alpha 1/2 Chain (COL4A1/A2) disorders	Collagen Type IV Alpha 1 Chain (COL4A1) Collagen Type IV Alpha 2 Chain (COL4A2)	Stroke; Eye defects; Migraine; Cardiac arrhythmia; Hemolytic anemia.

**Table 4:** Rarely mutant genes reported in HM^51,53^

Rarely mutant genes reported in HM
• Proline-rich transmembrane protein 2 (PRRT2)
• Paroxysmal Nonkinesigenic Dyskinesia (PNKD)
• Solute Carrier Family 2 Member 1 (SLC2A1)
• Solute Carrier Family 1 Member 3 (SLC1A3)
• Solute Carrier Family 4 member 4 (SLC4A4)

Studies on the genome-wide associations may be a successful strategy toward the identification of genes that enhance the susceptibility to migraine.

## 6. Treatment

The treatment of migraine-induced infarction is the same as for a typical infarction, including aspirin, which is a blood thinner to prevent clot formation, aspirin plus dipyridamole, warfarin for patients with atrial fibrillation, carotid endarterectomy, clopido-grel, and tissue plasminogen (tPA), which can breakdown clots. The treatment options of migraine-induced stroke have been listed in the Table 5^54^.

**Table 5:** Treatment options for migraine induced infarction^54^

Treatment options of migraine induced infarction
• Aspirin
• Aspirin plus dipyridamole
• Warfarin for patients with atrial fibrillation
• Carotid endarterectomy
• Clopidogrel
• Tissue plasminogen (tPA)

## 7. Directions for future research

Identification of the unique signaling pathways that regulate the brain’s susceptibility to the development of cortical spread depression may create opportu-nities for therapeutic modulation of those pathways.

Studies have shown that antiepileptic drugs may cause prevention of migraine and cortical spreading disorder^55-57^. However, in patients whose cerebral cortex has been acutely injured, these medications may have unpredictable effects resulting in the development of cortical spreading depression and even seizures in the presence of antiepileptic drugs (even in high doses), that in normal circumstances can decrease neuronal excitability^58^. There is a need for further studies to investigate whether a change in the antiepileptic medication used (typically phenytoin) may prove effective in reducing the susceptibility to cortical spreading depression initiation^58^.

## 8. Conclusion

Migrainous infarction is a very rare pathogenic event encountered in clinical practice. The exact mechanisms responsible for the migraine-induced stroke remains unclear. However, cortical spreading depression corresponds to the neurological symptoms observed in the disease. The people who are migraineurs should be advised to control other risk factors for stroke, such as diabetes, obesity and hypertension, etc. The patients with recurrent attacks of migraine with aura should be suspected of cerebral ischemia, and neuroimaging should be performed for early diagnosis and prevention of complications.
